# Intralesional Interferon Alfa‐2b Versus Cryotherapy for Common Warts on the Hands and Feet: A Randomized, Open‐Label, Controlled Trial

**DOI:** 10.1111/jocd.70740

**Published:** 2026-02-15

**Authors:** Yi Jin, Xiaoming Shen

**Affiliations:** ^1^ Department of Dermatology Suzhou Ninth People's Hospital (Suzhou Ninth Hospital Affiliated to Soochow University) Suzhou China

**Keywords:** adverse reactions, common warts, efficacy, hands and feet, injection therapy, interferon

## Abstract

**Objective:**

To evaluate the clinical efficacy of interferon injection therapy for common warts on the hands and feet and to analyze its associated adverse reactions.

**Methods:**

A total of 326 patients with common warts located on the hands and feet who were treated at our hospital between January 2024 and February 2025 were enrolled. Using a block randomization method, the patients were divided into a control group (*n* = 163, treated with cryotherapy) and an observation group (*n* = 163, treated with intralesional interferon alfa‐2b injection). The two groups were compared in terms of clinical efficacy, number, thickness, and diameter of warts, immune function, quality of life, adverse reactions, and recurrence rates.

**Results:**

After treatment, the number, thickness, and diameter of warts decreased in both groups, with significantly greater reductions in the observation group. Post‐treatment levels of cluster of differentiation 3 positive (CD3^+^), cluster of differentiation 4 positive (CD4^+^), the CD4^+^/CD8^+^ ratio, interleukin‐2 (IL‐2), interleukin‐6 (IL‐6), immunoglobulin E (IgE), and interferon‐gamma (IFN‐γ) increased, whereas cluster of differentiation 8 positive (CD8^+^) decreased in both groups. The observation group exhibited higher levels of CD3^+^, CD4^+^, CD4^+^/CD8^+^, IL‐2, IL‐6, IgE, and IFN‐γ, and lower CD8^+^ levels compared with the control group. Scores for all dimensions and total scores of the Dermatology Life Quality Index (DLQI) decreased after treatment in both groups, with significantly lower scores in the observation group.

**Conclusion:**

Interferon injection therapy demonstrates clinical efficacy in the treatment of common warts on the hands and feet.

## 1 Introduction

Common warts are benign hyperplastic skin lesions caused by infection with the human papillomavirus (HPV). They frequently occur on friction‐prone areas such as the hands and feet and have a high global incidence, particularly among children and adolescents, whose prevalence rates are significantly higher than those of adults [1]. Warts on the hands and feet not only affect appearance but can also cause pain‐induced functional impairment, severely diminishing patients' quality of life [2]. Currently, a wide range of clinical treatment options are available, including physical therapies (such as cryotherapy, laser ablation, and microwave therapy), chemical cauterization, topical medications, and immunomodulatory approaches. Among these, liquid nitrogen cryotherapy is a commonly used physical treatment due to its simplicity and low cost; however, it is often associated with adverse effects such as pain, blistering, and pigmentary changes, and it exhibits a relatively high recurrence rate for multiple or recalcitrant warts [3]. In recent years, interferon has been increasingly applied in the local injection therapy of viral warts because of its broad‐spectrum antiviral and immunomodulatory properties. Its primary mechanism involves inhibiting HPV replication and enhancing local cellular immunity to promote wart regression [4]. Several studies have reported that interferon shows therapeutic potential for recurrent or refractory warts; however, treatment is often accompanied by mild and reversible adverse reactions such as injection‐site pain, erythema, and influenza‐like symptoms (including fever, fatigue, and myalgia). The safety and tolerability of this therapy still warrant systematic evaluation [5]. Although previous research has explored the application of interferon in HPV‐related lesions, large‐sample randomized controlled trials (RCTs) focusing specifically on common warts of the hands and feet remain scarce [6, 7] Existing studies have primarily concentrated on condyloma acuminatum, with limited dedicated analyses of common warts and few direct comparative studies among different treatment modalities. Consequently, high‐level evidence‐based support is still lacking. Therefore, this randomized controlled trial was conducted to systematically evaluate the efficacy and safety of intralesional interferon alfa‐2b compared to standard cryotherapy for common warts on the hands and feet.

## 2 Materials and Methods

### 2.1 General Information

The sample size was calculated using the following formula: $N=\frac{P_{1}\left(1-P_{1}\right)+P_{2}\left(1-P_{2}\right)}{{\left(P_{1}-P_{2}\right)}^{2}}\times {\left(u_{\frac{\alpha }{2}}+u_{\beta }\right)}^{2}$. Based on the results of a previous study [8], the total effective rate in the study group was 92%, while that in the control group was 84%. Using these parameters (P₁ = 0.92, P₂ = 0.84, *α* = 0.05, *β* = 0.1), the calculated sample size was: $N=\frac{0.92\times \left(1-0.92\right)+0.84\times \left(1-0.84\right)}{{\left(0.92-0.84\right)}^{2}}\times {\left(1.96+1.28\right)}^{2}$ = 341. A total of 341 participants were initially enrolled. After 15 dropouts during the study period, 326 participants were included in the final analysis.

A total of 326 patients diagnosed with common warts on the hands and feet and treated at our hospital between January 2024 and February 2025 were enrolled. Inclusion criteria: (1) clinical diagnosis of common warts (verruca vulgaris) located on the dorsal aspect of the hands or feet [9]; (2) age ≥ 18 years; (3) no antiviral or physical therapy received within the previous 2 months; and (4) provision of signed informed consent. Exclusion criteria: (1) presence of severe organic diseases (e.g., cardiac, hepatic, or renal failure, or malignancy); (2) concomitant autoimmune disease, immunodeficiency, or current use of immunosuppressive agents; (3) known hypersensitivity to interferon preparations or a history of severe allergic reactions; (4) tendency to form keloids or residual scarring from previous treatments; or (5) pregnancy or lactation.

Using a block randomization method, the 341 participants were divided into 86 blocks (block length = 4). Random sequences were generated in Microsoft Excel, and participants within each block were assigned in a 1:1 ratio to either the control group or the observation group. The allocation scheme was sealed and maintained by an independent individual. Given the fundamentally different nature of the two procedures (injection vs. physical ablation), blinding of the participants and the treating clinicians was not feasible. Therefore, this was an open‐label trial. However, to minimize bias, the primary outcome assessor (a dermatologist who evaluated wart clearance and counted lesions from standardized photographs) and the statistician performing the data analysis were blinded to the group allocation.

This study was approved by the institutional ethics committee. All procedures were conducted in accordance with the ethical standards of the institutional and national research committees and with the 1964 Helsinki Declaration and its later amendments.

### 2.2 Treatment Methods

Liquid nitrogen cryotherapy was administered using a cryospray or cotton‐tip applicator. The lesion and a surrounding 1–2‐mm margin of healthy skin (the “freezing halo”) were frozen until a solid ice ball formed and was maintained for 5–10 s depending on wart thickness. Two freeze–thaw cycles were performed per session. Sessions were repeated every 2–3 weeks. The maximum number of sessions allowed per wart was 6. The median number of sessions actually received was 3 (range: 2–6). Post‐procedure care included disinfection and a sterile dressing. Recombinant human interferon alfa‐2b (Intron A, Merck & Co. Inc., Kenilworth, NJ, USA) was used. The lyophilized powder (3 million International Units [MIU] per vial) was reconstituted with 1 mL of sterile water for injection to yield a concentration of 3 MIU/mL. Using a 1‐mL insulin syringe with a 30‐gauge needle, the interferon solution was injected intralesionally into the base of each wart until slight blanching was observed. The injection volume per wart ranged from 0.1 to 0.3 mL, depending on wart size, with a maximum total dose of 1.5 MIU (0.5 mL of the reconstituted solution) per treatment session. Injections were administered once per week. The treatment course consisted of 4 weekly injections. If the wart persisted, a second course of up to 4 injections could be administered after a 4‐week observation period. The median number of injection sessions received was 4 (range: 4–8). No local anesthesia was routinely used prior to injection.

### 2.3 Observation Indicators

The pre‐specified primary outcome of this study is the “Treatment Success Rate,” an objective composite endpoint designed to accurately measure the core therapeutic efficacy. It is explicitly defined as achieving either of the following two objective criteria for the evaluated target wart(s) at the end of the treatment phase/course: (1) complete clearance: 100% shedding of the target wart(s) with full restoration of normal local skin texture and color; or (2) markedly effective: a reduction of greater than 70% in the surface area of the target wart(s) compared to baseline, based on standardized planimetric measurement. To ensure objectivity and reproducibility of the assessment, a rigorous evaluation protocol was established. High‐resolution digital photographs of the target wart(s) were taken under consistent lighting, angle, and with an included scale by personnel not involved in treatment delivery, both pre‐treatment and 4 weeks post‐treatment. Subsequently, all photographs were anonymized and randomized. Two dermatologists, completely blinded to group allocation, independently measured the wart area using image analysis software. The final result was the average of the two measurements. In case of disagreement between the two assessors regarding the attainment of the > 70% reduction threshold, a third senior blinded assessor made the final adjudication. For patients with multiple warts, the largest or most representative single wart was designated as the “target wart” for this assessment.

The key secondary outcomes aim to provide complementary and descriptive analyses of efficacy from different dimensions, their evaluation also grounded in objective and quantifiable methods. The first key secondary outcome is the “Efficacy evaluation criteria [9]”. This classification is directly derived from the measurement data of the primary outcome described above, offering a more granular view of efficacy distribution for descriptive analysis. Patients are objectively categorized into one of four grades: (1) cured: complete clearance of warts, restoration of normal skin, and no new lesions. (2) markedly effective: wart shedding area > 70% and local tissue thinning. (3) improved: wart shedding area between 30% and 70% with local tissue thinning. (4) ineffective: wart shedding area < 30%, no change in lesion, or new lesion formation. The second key secondary outcome is the Wart scoring criteria, which integrates a multi‐dimensional objective assessment of lesion burden. This specifically encompasses: an “exact total count of all visible warts” performed by a separate independent blinded assessor based on standardized photographs; and precise measurements of the “maximum diameter” and “maximum thickness” (estimated via image shadow contrast or lateral view reference) of the target wart. Finally, the wart count, maximum diameter, and maximum thickness are converted into standardized scores according to a predefined scale and summed to generate a composite score for each patient. The specific scoring rules are as follows: (1) number of warts: 0 = 0 points; 1–2 = 2 points; 3–5 = 4 points; ≥ 6 = 6 points. (2) diameter of warts: none = 0 points; ≤ 2 mm = 2 points; 2–5 mm = 4 points; ≥ 5 mm = 6 points. (3) Thickness of warts: flush with epidermis = 0 points; < 1 mm = 2 points; 1–2 mm = 4 points; ≥ 2 mm = 6 points.

Other secondary outcomes are employed to comprehensively evaluate the holistic impact and safety profile of the treatments. These include: (1) immune function: before and after treatment, T lymphocyte subsets were measured using flow cytometry, including cluster of differentiation 3 positive (CD3^+^), cluster of differentiation 4 positive (CD4^+^), and cluster of differentiation 8 positive (CD8^+^) cells, and the CD4^+^/CD8^+^ ratio was calculated. Peripheral venous blood samples (3–5 mL) were collected and centrifuged at 1500–2000× *g* for 10 min to obtain serum. Levels of interleukin‐2 (IL‐2) and interleukin‐6 (IL‐6) were determined using enzyme‐linked immunosorbent assay (ELISA), and immunoglobulin E (IgE) was measured by chemiluminescent immunoassay. (2) Quality of life: The Dermatology Life Quality Index (DLQI) [10] was used to assess quality of life before and after treatment. The DLQI comprises five dimensions: symptoms, feelings, daily activities, work/study, and interpersonal relationships. Each item is scored from 0 to 3, with higher scores indicating poorer quality of life. (3) Adverse reactions and recurrence: Actively solicited and recorded at each visit using a standardized questionnaire. Local AEs (pain, blister, infection) and systemic AEs (fever > 38°C, fatigue, myalgia, headache) were graded as mild, moderate, or severe. Any laboratory abnormalities were also recorded. (4) Recurrence: defined as the appearance of a new wart at the site of a previously completely cleared lesion. Recurrence was assessed at 1, 3, and 6 months after the end of treatment. The cumulative recurrence rate at 6 months was calculated as the number of patients with any recurrence divided by the number of patients who achieved complete clearance in that group.

### 2.4 Statistical Analysis

Data were analyzed using SPSS Statistics version 22.0 (IBM Corp., Armonk, NY, USA). Quantitative data following a normal distribution were expressed as mean ± standard deviation (SD). Independent‐samples *t*‐tests were used for comparisons between groups, and paired *t*‐tests for within‐group comparisons. Non‐normally distributed data were expressed as median (interquartile range) (*M* [P25–P75]) and compared using the Wilcoxon rank‐sum test (between groups) or paired Wilcoxon signed‐rank test (within groups). Qualitative data were presented as frequencies and percentages (*n* [%]), and compared using the chi‐squared (*χ* ^2^) test. Unless otherwise specified, the significance level (*α*) was set at 0.05, and *p* < *α* was considered statistically significant.

## 3 Results

### 3.1 Participant Flow Diagram

A total of 341 patients were assessed for eligibility, all of whom were randomized into two groups: the observation group (interferon therapy, *n* = 170) and the control group (cryotherapy, *n* = 171). All patients received their allocated interventions (Figure S1).

During the follow‐up period, 15 patients discontinued the study. In the observation group, 7 patients discontinued (6 due to loss to follow‐up and 1 due to personal reasons). In the control group, 8 patients discontinued (3 due to loss to follow‐up, 4 due to treatment discomfort, and 1 due to personal reasons) (Figure S1).

Ultimately, 326 patients completed the study (163 in each group) and were included in the intention‐to‐treat analysis for efficacy. The safety analysis included all patients who received at least one treatment session (*n* = 341) (Figure S1).

### 3.2 Comparison of Baseline Data

Patients were divided into a control group (*n* = 163, treated with cryotherapy) and an observation group (*n* = 163, treated with interferon injection). There were no statistically significant differences in baseline characteristics between the two groups (*p* > 0.05) (Table 1).

**TABLE 1:** Comparison of baseline data between two groups ($\bar{x}\pm s$ [*n*, %]).

Baseline data	Observation group	Control group	*t/χ* ^2^ value	*p*
Age (years)	52.77 ± 15.61	54.68 ± 16.34	1.078	0.282
Sex				
Male	87 (53.37)	79 (48.47)	0.786	0.375
Female	76 (46.63)	84 (51.53)		
BMI (kg/m^2^)	24.57 ± 1.74	24.36 ± 1.88	1.045	0.297
Wart location				
Hand	24 (14.72)	37 (22.70)	5.541	0.063
Foot	123 (75.46)	118 (72.39)		
Hand and feet	16 (9.82)	8 (4.91)		
Courses (months)				
< 3	20 (12.27)	31 (19.02)	5.024	0.081
3–6	36 (22.09)	44 (26.99)		
> 6	107 (65.64)	88 (53.99)		
Previous treatment history	52 (31.90)	40 (24.54)	2.181	0.140

### 3.3 Comparison of Clinical Efficacy

The treatment success rate was 55.21% (90/163) in the observation group and 49.08% (80/163) in the control group; however, this difference was not statistically significant (risk ratio [RR] = 1.131, 95% CI: 0.910 to 1.413; *p* = 0.268). Conversely, the rate of ineffective treatment was significantly lower in the observation group (7.98% [13/163]) than in the control group (18.40% [30/163]) (RR = 0.570, 95% CI: 0.347 to 0.866; *p* = 0.005). Details of the efficacy comparisons are shown in Table 2.

**TABLE 2:** Comparison of clinical efficacy between two groups (*n*, %).

Groups	Cases	Cured	Markedly effective	Improved	Ineffective	Treatment success
Observation group	163	47 (28.83)	43 (26.38)	60 (36.81)	13 (7.98)	90 (55.21)
Control group	163	43 (26.38)	37 (22.70)	53 (32.52)	30 (18.40)	80 (49.08)
*χ* ^2^ value	—	0.496	0.772	0.815	2.782	1.109
P	—	0.620	0.440	0.415	0.005	0.268
Risk ratio	—	1.062	1.102	1.098	0.570	1.131
95% CI	—	0.828–1.331	0.854–1.382	0.873–1.363	0.347–0.866	0.910–1.413

### 3.4 Comparison of Wart Number, Thickness, and Diameter

Compared with pre‐treatment values, the number, thickness, and diameter of warts in both groups were significantly reduced after treatment (*p* < 0.05) (Figure S2). Moreover, post‐treatment values in the observation group were significantly lower than those in the control group (*p* < 0.05) (Table 3).

**TABLE 3:** Comparison of number, thickness, and diameter of warts between two groups (*M* [P25–P75]).

Groups	Case	Number of warts (pcs)		Wart thickness (mm)		Wart diameter (mm)	
		Before treatment	After treatment	Before treatment	After treatment	Before treatment	After treatment
Observation group	163	4.00 (2.00–6.00)	2.00 (0.00–4.00)^a^	4.00 (2.00–6.00)	1.00 (0.00–2.00)^a^	4.00 (3.00–6.00)	1.00 (0.00–2.00)^a^
Control group	163	4.00 (2.00–6.00)	3.00 (2.00–4.00)^a^	4.00 (2.00–6.00)	1.00 (0.00–2.00)^a^	4.00 (3.00–6.00)	2.00 (0.00–3.00)^a^
*Z*		−0.404	−3.087	−0.968	−2.243	−0.210	−8.102
*p*		0.686	0.002	0.333	0.025	0.833	< 0.001

*Note:* Compared to before treatment, ^a^ *p* < 0.05.

### 3.5 Comparison of Immune Function

After treatment, both groups showed significant increases in CD3^+^, CD4^+^, CD4^+^/CD8^+^, IL‐2, IL‐6, IgE, and IFN‐γ levels, along with a decrease in CD8^+^ levels (*p* < 0.05). Compared with the control group, the observation group exhibited higher post‐treatment levels of CD3^+^, CD4^+^, CD4^+^/CD8^+^, IL‐2, IL‐6, IgE, and IFN‐γ, and lower levels of CD8^+^ (*p* < 0.05) (Table 4).

**TABLE 4:** Comparison of immune function between two groups ($\bar{x}\pm s$).

Groups	Cases	CD3^+^ (%)		CD4^+^ (%)		CD8^+^ (%)		CD4^+^/CD8^+^	
		Before treatment	After treatment	Before treatment	After treatment	Before treatment	After treatment	Before treatment	After treatment
Observation group	163	63.58 ± 5.49	70.83 ± 3.81^a^	38.12 ± 4.28	45.83 ± 2.67^a^	29.15 ± 3.42	22.61 ± 2.15^a^	1.32 ± 0.14	2.04 ± 0.15^a^
Control group	163	63.77 ± 5.41	67.86 ± 4.35^a^	38.74 ± 4.33	42.15 ± 3.38^a^	29.09 ± 3.59	24.85 ± 2.81^a^	1.34 ± 0.16	1.71 ± 0.16^a^
*t* value		0.315	6.570	1.286	10.907	0.142	8.054	1.625	19.135
*p*		0.753	< 0.001	0.199	< 0.001	0.887	< 0.001	0.105	< 0.001

Groups	Cases	IL‐2 (ng/mL)		IL‐6 (ng/mL)		IgE (IU/mL)		INF‐γ (ng/mL)	
		Before treatment	After treatment	Before treatment	After treatment	Before treatment	After treatment	Before treatment	After treatment
Observation group	163	2.61 ± 0.78	4.5 ± 1.33^a^	1.86 ± 0.55	3.49 ± 0.93^a^	66.64 ± 11.93	100.59 ± 7.34^a^	27.44 ± 7.48	34.84 ± 9.39^a^
Control group	163	2.72 ± 0.79	3.53 ± 1.13^a^	1.91 ± 0.64	2.67 ± 0.66^a^	68.15 ± 11.74	91.85 ± 7.86^a^	28.34 ± 7.61	30.90 ± 8.70^a^
*t* value		1.262	7.109	0.764	9.228	1.151	10.376	1.077	3.929
*p*		0.208	< 0.001	0.445	< 0.001	0.250	< 0.001	0.282	< 0.001

*Note:* Compared to before treatment, ^a^ *p* < 0.05.

### 3.6 Comparison of Quality of Life

Following treatment, DLQI scores across all dimensions and total scores were significantly reduced in both groups compared with pre‐treatment levels (*p* < 0.05). Furthermore, the observation group demonstrated significantly lower DLQI scores than the control group after treatment (*p* < 0.05) (Table 5).

**TABLE 5:** DLQI scores across all dimensions and total scores ($\bar{x}\pm s$).

Groups	Cases	Symptom		Feeling the situation		Daily activities	
		Before treatment	After treatment	Before treatment	After treatment	Before treatment	After treatment
Observation group	163	2.44 ± 0.5	1.09 ± 0.28^a^	2.66 ± 0.57	1.04 ± 0.20^a^	2.45 ± 0.50	1.36 ± 0.48^a^
Control group	163	2.35 ± 0.49	1.91 ± 0.29^a^	2.69 ± 0.55	1.67 ± 0.47^a^	2.47 ± 0.50	1.53 ± 0.50^a^
*t* value		1.569	25.991	0.594	15.541	0.443	3.275
*p*		0.118	< 0.001	0.553	< 0.001	0.658	0.001

Groups	Cases	Work and study		Interpersonal relationships		DLQI total score	
		Before treatment	After treatment	Before treatment	After treatment	Before treatment	After treatment
Observation group	163	2.32 ± 0.47	1.43 ± 0.50^a^	2.54 ± 0.50	1.02 ± 0.16^a^	12.40 ± 1.49	5.94 ± 1.11^a^
Control group	163	2.39 ± 0.51	1.63 ± 0.66^a^	2.61 ± 0.49	1.53 ± 0.50^a^	12.52 ± 1.51	8.27 ± 1.60^a^
*t* value		1.352	3.139	1.345	12.251	0.739	15.270
*p*		0.177	0.002	0.180	< 0.001	0.460	< 0.001

*Note:* Compared to before treatment, ^a^ *p* < 0.05.

### 3.7 Comparison of Adverse Reactions

The overall incidence of adverse reactions in the observation group was 9.20%, significantly lower than that in the control group (16.56%) (*p* < 0.05) (Table 6).

**TABLE 6:** Comparison of adverse reactions between two groups (*n*, %).

Groups	Cases	Blister (blood) blister	Pain	Infection	Any systemic flu‐like symptom	Total adverse reactions
Observation	163	2 (1.23)	2 (1.23)	2 (1.23)	9 (5.52)	15 (9.20)
Control group	163	9 (5.59)	7 (4.29)	9 (5.52)	2 (1.23)	27 (16.56)
*χ* ^2^ value		2.168	1.690	2.147	2.147	1.984
*p*		0.030	0.091	0.032	0.032	0.047
95% CI		0.100–0.936	0.124–1.089	0.100–0.942	1.058–2.052	0.436–0.996

### 3.8 Comparison of Recurrence Rates

At the 6‐month follow‐up, the cumulative recurrence rate was 26.67% (24/90) in the observation group, which was significantly lower than that in the control group (70.00%, 56/80) (*χ* ^2^ = 5.650, *p* < 0.001). At specific time points: at 1 month post‐treatment, the recurrence rate was 0.00% (0/90) in the observation group, significantly lower than 11.25% (9/80) in the control group (*p* = 0.001); at 3 months post‐treatment, there was no statistically significant difference between the two groups (13.33% vs. 21.25%, *p* = 0.171); at 6 months post‐treatment, the recurrence rate was 13.33% (12/90) in the observation group, significantly lower than 37.50% (30/80) in the control group (*p* < 0.001) (Table 7).

**TABLE 7:** Comparison of recurrence rates between two groups (*n*, %).

Groups	Case	1 month after treatment	3 months after treatment	6 months after treatment	Cumulative recurrence
Observation	90	0 (0.00)	12 (13.33)	12 (13.33)	24 (26.67)
Control group	80	9 (11.25)	17 (21.25)	30 (37.50)	56 (70.0)
*χ* ^2^ value	—	3.270	1.370	3.647	5.650
*p*	—	0.001	0.171	< 0.001	< 0.001
95% CI	—	0.000–0.538	0.452–1.113	0.277–0.735	0.282–0.573

## 4 Discussion

This randomized controlled study demonstrated that, for common warts on the hands and feet, local interferon injection achieved a significantly higher overall clinical efficacy than conventional cryotherapy, while also exhibiting notable advantages in reducing post‐treatment recurrence and minimizing adverse events. This key finding suggests that interferon injection, as an etiology‐targeted biological therapy, holds substantial clinical value in the management of common warts. While cryotherapy is known to exert some immunostimulatory effects alongside tissue destruction, the immunomodulatory action of interferon is a direct and central therapeutic mechanism [11, 12, 13]. To our knowledge, this is one of the first large‐scale, randomized controlled trials to systematically compare the comprehensive effects of interferon therapy versus cryotherapy on wart clearance, immune function, inflammatory cytokine levels, and quality of life in patients with hand and foot common warts, thereby providing more robust evidence‐based support for its clinical application. From a clinical standpoint, interferon therapy offers dual benefits: on one hand, it enhances viral clearance through activation of host antiviral immunity; on the other hand, its targeted mechanism minimizes damage to surrounding healthy tissue. Furthermore, the interferon‐treated group exhibited greater improvement in quality‐of‐life scores compared with the cryotherapy group, which may be attributed to less pain, faster recovery, and minimal interference with daily activities. It is worth noting that traditional cryotherapy is associated with a higher recurrence rate in our study, suggesting that while it may trigger an immune response, interferon therapy might induce a more robust or sustained immunologic clearance of latent HPV infection.

Common warts are benign hyperplastic skin lesions caused by infection with HPV, commonly occurring on friction‐prone areas such as the hands and feet [14]. The remarkable efficacy of interferon therapy for common warts stems from its multifaceted mechanism of action. While cryotherapy primarily induces tissue necrosis and may subsequently stimulate an immune response against viral antigens released from destroyed cells [11], interferon—a key cytokine—exerts both direct antiviral and active immunomodulatory effects as its primary mode of action [15]. Regarding the direct antiviral mechanism, interferon activates intracellular antiviral pathways to effectively inhibit HPV replication [16]. Specifically, upon binding to specific receptors on target cell membranes, interferon activates the JAK–STAT signaling pathway and induces the expression of hundreds of interferon‐stimulated genes (ISGs), whose products interfere with viral nucleic acid transcription and translation, thereby blocking viral protein synthesis and suppressing HPV replication and spread at its source. In terms of immune regulation, HPV employs various strategies to evade immune surveillance, such as downregulating antigen‐presenting molecules and suppressing interferon signaling pathways. Interferon can effectively reverse this local immunosuppressive microenvironment induced by HPV infection [17]. In this study, patients treated with interferon showed significantly increased proportions of CD3+ and CD4+ T cells, an elevated CD4+/CD8+ ratio, and upregulated levels of IFN‐γ and IL‐2, findings that directly reflect its active and potent immunoregulatory role. Interferon activates antigen‐presenting cells such as dendritic cells to enhance viral antigen processing and presentation; it promotes Th1‐type immune responses, boosts cytotoxic T lymphocyte and natural killer cell activity against infected cells, and modulates cytokine networks to create a local environment unfavorable to viral persistence [18, 19].

The immunomodulatory effects observed in our study align with the proposed mechanism of action for intralesional interferon in wart treatment. Previous studies on both genital and non‐genital warts have suggested that interferon's therapeutic effect extends beyond direct antiviral activity to include enhancement of cell‐mediated immunity [20, 21, 22]. For instance, immunohistochemical analyses of wart tissue before and after interferon treatment have shown increased infiltration of CD4+ T lymphocytes and alterations in local cytokine profiles [23], supporting the systemic immune changes we observed in peripheral blood.

The results of this study are consistent with those of Welander CE et al. [24], who also confirmed the reliable efficacy and low recurrence rate of interferon therapy for genital warts. However, compared with most previous studies, this work presents several novel contributions and theoretical advancements. In terms of study design, although prior studies have explored intralesional interferon for condyloma acuminatum or verruca plantaris [20, 25], this study, employing a block‐randomized controlled design, provides a direct head‐to‐head comparison between interferon monotherapy and the standard cryotherapy for common warts on both hands and feet, addressing an important evidence gap regarding their relative effectiveness and impact on patient‐centered outcomes. Warts in these regions pose therapeutic challenges due to their unique anatomical characteristics and continuous mechanical friction, often resulting in higher recurrence rates; our findings provide a targeted solution for this clinical problem. Regarding outcome measures, previous studies mainly assessed morphological improvements and short‐term recurrence rates, with limited attention to immunological parameters and quality of life. In contrast, the present study evaluated not only morphological indices such as the number, thickness, and diameter of warts, but also systematically analyzed immune parameters, including T‐cell subsets and cytokine profiles, while introducing the DLQI to quantify quality‐of‐life improvement. These comprehensive data offer stronger experimental support for elucidating the mechanisms underlying interferon therapy.

The clinical value of interferon injection therapy for common warts lies in its multidimensional advantages encompassing efficacy, safety, prognosis, and quality‐of‐life improvement. In terms of therapeutic effect, interferon demonstrates a “curative‐from‐the‐root” property by fundamentally controlling disease progression. Although cryotherapy can also elicit an immune response, interferon's combined antiviral and active immunomodulatory actions not only eliminate existing warts but also more effectively target adjacent latent common adverse effects, which is believed to contribute to its lower recurrence rates observed in this and previous studies [21, 22]. This advantage is particularly significant for patients with multiple or recalcitrant warts. Regarding safety, interferon therapy exhibits a lower incidence of adverse events compared with cryotherapy. The latter causes tissue destruction through freezing, often resulting in pain, blistering, or hemorrhagic bullae, and may lead to pigmentary alterations or scarring. In contrast, intralesional interferon injection is generally well‐tolerated, with the most common adverse effects being transient local reactions (pain, erythema, swelling) and self‐limiting flu‐like symptoms (fever, fatigue, myalgia), which rarely cause permanent structural damage [26, 27] This safety profile is especially relevant for exposed sites such as the hands and feet, directly influencing patients' treatment choices and compliance. In terms of prognosis and quality of life, interferon therapy offers multiple benefits: by reducing recurrence, it decreases repeated clinic visits and economic burden while alleviating psychological stress caused by disease uncertainty; in addition, because interferon promotes gradual wart regression rather than destructive detachment, it better preserves the normal appearance and function of the skin, facilitating social and daily reintegration. DLQI assessment in this study confirmed that patients in the interferon group experienced significantly greater quality‐of‐life improvement than those in the cryotherapy group, reflecting comprehensive gains in symptom control, functional recovery, and psychosocial adaptation.

Despite these promising results, several limitations should be acknowledged. First, the sample size was relatively limited, and the study population did not fully represent different age groups, immune statuses, or wart types. Additionally, it should be noted that no upper limit was defined for the number of warts included in the study. Common warts are more prevalent among children and adolescents, whereas elderly or immunocompromised individuals may exhibit distinct therapeutic responses and prognostic patterns. Future studies should include larger, more diverse populations and perform subgroup analyses to clarify the differential efficacy of interferon therapy across demographic and clinical subgroups. Second, the follow‐up duration was insufficient to evaluate long‐term recurrence and the persistence of immune responses. Because recurrences often occur several months to 1 year after treatment, the short follow‐up period may have underestimated recurrence rates. Extended follow‐up of at least 1 year is recommended to comprehensively assess long‐term outcomes and immune memory sustainability. Third, standardized protocols for interferon injection, such as dosage, frequency, and treatment duration, have not yet been established, potentially contributing to variability in therapeutic response. In contrast to cryotherapy, for which protocols are well defined, the optimal regimen for interferon therapy requires further investigation through dose‐gradient and interval‐optimization studies to establish more precise clinical guidelines. Finally, while this study demonstrated associations between interferon therapy and improved immune indicators, the precise molecular mechanisms remain to be elucidated. Future research should explore the differential sensitivity of various HPV genotypes to interferon, as well as the influence of host genetic polymorphisms on interferon signaling. Incorporating HPV genotyping and molecular biomarker analysis could facilitate a more individualized therapeutic approach.

In conclusion, compared with conventional cryotherapy, local interferon injection for hand and foot common warts provides superior wart clearance, enhances systemic and local immune parameters, reduces recurrence risk, and improves quality of life, highlighting its comprehensive clinical advantages. Its therapeutic effects are primarily mediated through direct inhibition of HPV replication and activation of local cellular immune responses, embodying a “treating the root cause rather than the symptom” principle.

## Author Contributions

Yi Jin conceived and designed the research study, performed statistical analysis, acquired and analyzed the data, and wrote the initial draft of the manuscript. Xiaoming Shen provided expertise in the field of study, contributed to the interpretation of the data, and critically revised the manuscript for important intellectual content. All authors have read and approved the final manuscript and have participated sufficiently in the work to take public responsibility for appropriate portions of the content.

## Funding

The authors have nothing to report.

## Ethics Statement

This study was approved by the Human Ethics Committee of Suzhou Ninth People's Hospital (Suzhou Ninth Hospital Affiliated to Soochow University). The requirement for informed consent was waived because this study was a retrospective analysis of existing data and did not involve any intervention or identifiable personal information. All procedures were conducted in accordance with the ethical standards of the institutional and national research committees and with the 1964 Helsinki Declaration and its later amendments.

## Conflicts of Interest

The authors declare no conflicts of interest.

## Supporting information


**Data S1:** jocd70740‐sup‐0001‐FigureS1‐S2.docx.

## Data Availability

The datas used and/or analyzed during the current study are available from the corresponding author.
