# Supplementary material for: Intralesional Interferon Alfa‐2b Versus Cryotherapy for Common Warts on the Hands and Feet: A Randomized, Open‐Label, Controlled Trial
Source: J Cosmet Dermatol. 2026 Feb 15;25(2):e70740. doi: 10.1111/jocd.70740 (PMC12907598; doi:10.1111/jocd.70740)
Supplement: Supplementary file 1 — Data S1: jocd70740‐sup‐0001‐FigureS1‐S2.docx. [file JOCD-25-e70740-s001.docx]

Supplementary Figure 1 The CONSORT flow diagram


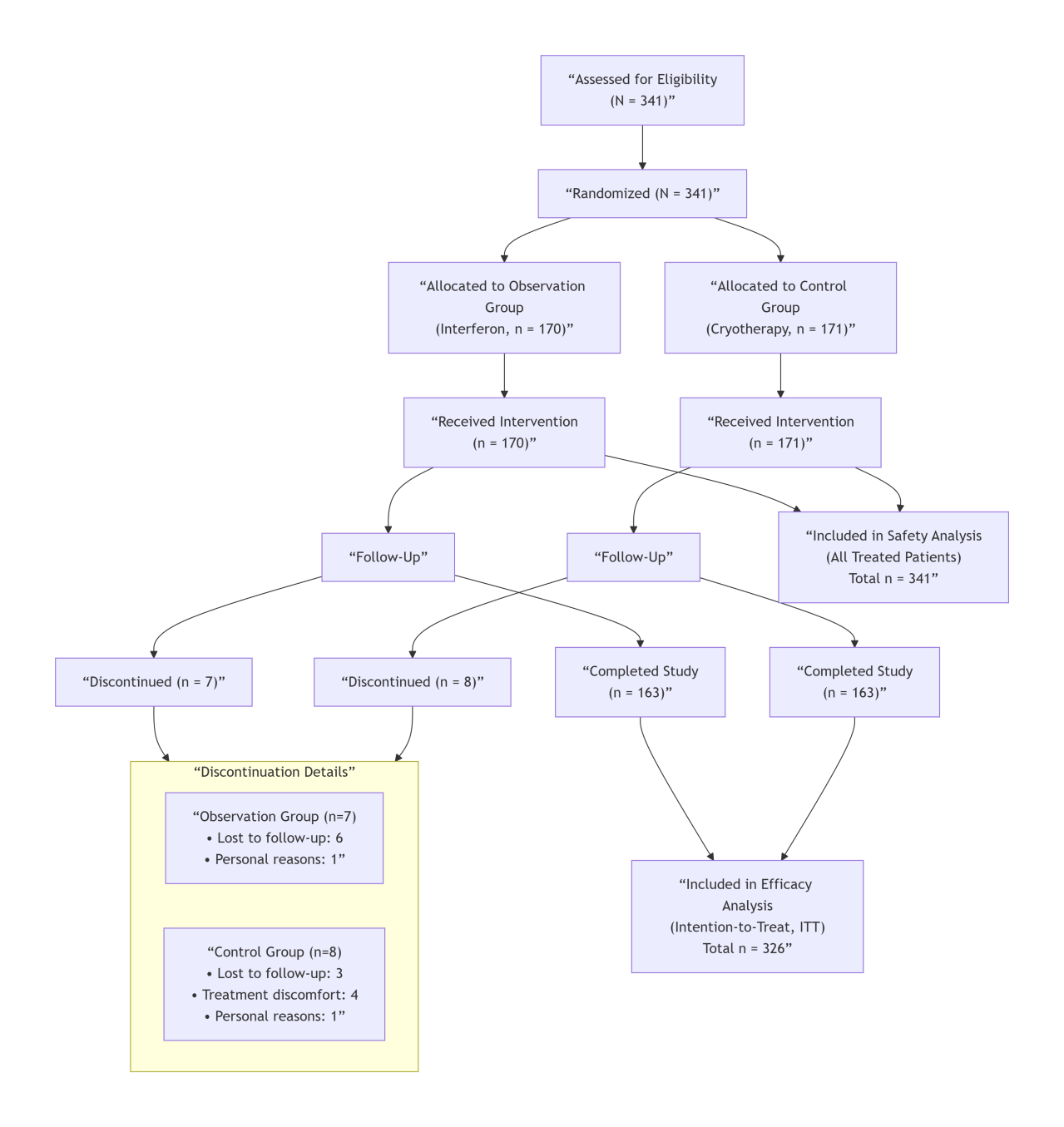


Supplementary Figure 2 Comparison of Wart Number, Thickness, and Diameter in Group Communication


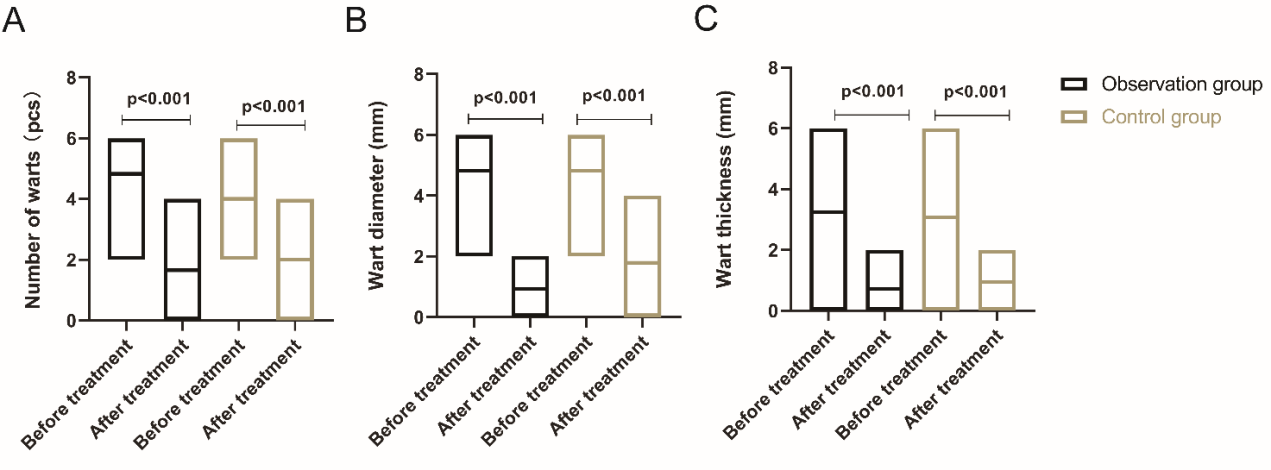


Note: (A) Changes in the number of warts within each group before and after treatment; (B) Changes in wart thickness within each group before and after treatment; (C) Changes in wart diameter within each group before and after treatment.
